# Temporal trends in biosecurity in Irish pig herds using a standardized scoring system

**DOI:** 10.1186/s13620-025-00289-0

**Published:** 2025-01-30

**Authors:** Carla Correia-Gomes, Bárbara Terezo, David Graham

**Affiliations:** 1 Animal Health Ireland, 4-5 The Archways, Carrick On Shannon, Co. Leitrim N41 WN27 Ireland; 2 Teagasc, Pig Development Department, Animal & Grassland Research and Innovation Centre,Teagasc, Moorepark, Fermoy Co. Cork, P61 C997 Ireland

**Keywords:** Biosecurity, Pigs, Ireland

## Abstract

**Background:**

Biosecurity measures are crucial to the introduction and spread of pathogens both within and between farms. External biosecurity focuses on preventing pathogens from entering or leaving the farm, while internal biosecurity aims to limit or stop the spread of pathogens within the farm. Implementing biosecurity measures not only protects animals from disease but also has positive effects on productivity, welfare and farm profitability. By reducing the need for antimicrobials, biosecurity measures also contribute to combating antimicrobial resistance. To assess and improve biosecurity, tools like Biocheck.UGent have been developed. In Ireland, the Biocheck.UGent tool has been widely used in pig farms since 2018. The aims of this study were firstly to assess temporal trends on biosecurity scores in Irish pig farms from 2018 to 2023 using the Biocheck.UGent tool and secondly to identify areas for improvement.

**Results:**

There was an increase in the number of annual assessments over the study period, with the majority of farms being assessed multiple times. Overall, external biosecurity scores were higher than internal scores. Improvements in the scores were observed over time for most of the subcategories for external and internal biosecurity and across the different farm types. Analysis of the subcategories within the scoring system revealed areas with lower scores, including disease management, cleaning and disinfection, and measures between compartments. Weaner-to-finisher farms tended to have lower scores compared to other farm types.

**Conclusions:**

While external biosecurity in Irish pig farms is generally high, there is room for improvement in internal biosecurity. This study highlights the importance of continuous efforts to improve biosecurity. The data obtained will aid in estimating the cost–benefit of implementing biosecurity measures, crucial for decision-making and better returns on investments.

**Supplementary Information:**

The online version contains supplementary material available at 10.1186/s13620-025-00289-0.

## Background

Biosecurity is paramount to the production of healthy animals. At the farm level, it includes all measures taken to minimise the risk of introduction and spread of pathogens. By taking these biosecurity measures and performing efficient management, on-farm animals are protected against both endemic and exotic diseases [1]. Biosecurity can be divided into external and internal components. External biosecurity focuses on the contact points of the farm with the outside world and aims to prevent pathogens from entering or leaving the farm. In an Irish context, this applies both to exotic diseases such as African Swine Fever, as well as to endemic diseases such as porcine reproductive and respiratory syndrome (PRRS) [15]. All measures taken to limit or stop the spread of pathogens within a farm are included within internal biosecurity [12].

The implementation of biosecurity measures has also been shown to have other positive effects. For example, in several studies with pigs, biosecurity showed a positive correlation with production traits such as daily growth [16] and the profitability of the farm [12, 17]. Along with this, the use of antimicrobials can be greatly reduced [12, 14], which, consequently, will reduce antimicrobial resistance (AMR; [8]).

Several tools have been developed for measuring biosecurity at farm level, either generically (e.g. including the Biocheck.UGent tools [https://biocheckgent.com/en]), or for specific diseases (e.g. COMBAT [https://www.prrs.com/disease-control/control/combat]).

As part of Ireland’s efforts to prevent the introduction of exotic diseases (such as African Swine Fever) and improve biosecurity at national level, as per the National Farmed Animal Biosecurity Strategy (2021–2024) [2], since 2018, the Biocheck.UGent tool has been widely used on Irish pig farms. The Targeted Advisory Service on Animal Health (TASAH) under the Rural Development Programme funded one free biosecurity assessment for commercial pig farms per calendar year.

The aim of this study was to: a) assess the biosecurity levels for the pig industry in Ireland, using the Biocheck.UGent assessment method, over time, from 2018 to 2023, and per production system; and b) identify areas for improvement.

## Methods

### Biosecurity scoring tool

Biocheck.UGent is a risk-based scoring system developed by the University of Ghent to evaluate the quality of on-farm biosecurity for a variety of species and production systems, including pigs, using an objective, weighted scoring system (https://www.biocheck.ugent.en/). For pig farms, external and internal biosecurity are each divided into several subcategories, which address different pathways of pathogen introduction/spread, including live animal movements, transport vehicles, people, materials and equipment, air, pests and feed (https://biocheckgent.com/en/about-biosecurity-pig). The external subcategories are: purchase of animals and semen (E1-Purchase), transport of animals, removal of manure/dead animals (E2-Transport), feed, water and equipment supply (E3-Feed), personnel and visitors (E4-Personnel), vermin/bird control (E5-Vermin), and environment and region (E6-Location). The internal subcategories are: disease management (I1-Disease), farrowing and suckling period (I2-Farrowing), nursery unit management (I3-Nursery), fattening unit management (I4-Fattening), measures between compartments and the use of equipment (I5-Compartments), and cleaning and disinfection (I6-C&D). I2 to I4 are only scored if there are breeding animals, weaners or fatteners, respectively, on the farm.

### Biosecurity assessment scheme

As part of Ireland's efforts to prevent the introduction of African Swine Fever in the country and improve biosecurity at national level, TASAH under the Rural Development Programme (2014–2020) funded one free biosecurity assessment for commercial pig farms (loosely defined as sending at least 200 pigs to slaughter per year or having 200 pigs in the farm) per calendar year. These assessments were done by private veterinary practitioners (PVPs). It was a pre-requisite of TASAH that all participating PVPs had to be trained on using the Biocheck.UGent tool before doing the assessments. The training also included aspects of communication and development of SMART (specific, measurable, attainable, realistic, time-bounded) recommendations.

### Biosecurity assessment implementation

PVPs would organise the visits with the farm owner/manager, do the assessment on farm (including a farm visit for visual inspection) following the Biocheck.UGent questionnaire, and provide a maximum of three SMART recommendations for improving biosecurity that were agreed with the farmer. They would then input the questionnaire answers (and get the BiocheckUgent weighted scores through an API) and recommendations in the Animal Health Ireland (AHI) Pig HealthCheck web system (since January 2022). AHI is a private–public partnership that since 2019 runs the Pig HealthCheck programme, which is co-funded by pig producers and the Department of Agriculture Food and the Marine (DAFM), with the aim of improving the profitability and sustainability of the Irish pig industry through improved animal health. The programme comprises five activities, of which biosecurity is one (https://animalhealthireland.ie/programmes/pig-healthcheck/introduction/). Central to the Pig HealthCheck Programme is its database that allows all data captured from the key programme activities to be linked and analysed. The database allows the creation of dashboards for each component of the programme and displays the farm data and benchmarks them against the performances of other herds and national averages (https://animalhealthireland.ie/programmes/pig-healthcheck/pig-healthcheck-database/).

### Farm types

Farm type was defined as follows: farrow-to-finish farms (farms that had breeding animals, weaners and finisher animals) (FTF), breeder farms (farms that had breeding animals but no weaners and finisher animals), farrow-to-weaner farms (farms that had breeding animals and weaners) (FTW), weaner farms (farms that only had weaners), weaner-to-finisher farms (farms that had weaners and finisher animals) (WTF), finisher farms (farms that only had finishers animals) (F). For the analysis breeder units were aggregated with farrow-to-weaner (FTW) units and weaners units were aggregated with weaner-to-finisher (WTF) units because there were only a few breeder and weaners units assessed (7 each).

### Data analysis

The number of non-breeding animals was calculated as the sum of the number of weaners and the number of finishers per farm. The number of animals was then transformed into a log10 scale.

Pearson correlation test was used to estimate the correlation between external and internal biosecurity scores per farm. A correlation coefficient of ≤ 0.35 was considered low, 0.36–0.67 was considered moderate, 0.68–0.89 was considered high and ≥ 0.90 was considered very high [18].

To detect if there were differences for external, internal and overall scores in relation to farm type or over the years, ANOVA analyses were conducted and if *p* < 0.05, Turkey post hoc tests were conducted. This was done by year and excluding 2018 results (due to the low number of assessments).

For the analysis of results over time for the same farms, a cohort of farms with at least three assessments was chosen from the study farms. The last three most recent assessments for that cohort were compared graphically. The assessments were presented as A (third last most recent assessment for the farm), B (second last most recent assessment for the farm) and C (most recent assessment for the farm) and therefore do not necessarily represent consecutive years for all farms in that cohort.

Summary statistics, such as counts and percentages were done for categorical variables, while measures of centrality (e.g. mean, median) and location (e.g. first quartile) were estimated for continuous variables. This was done in Rstudio using the packages plyr, tidyverse [20], tidyr [23], dplyr [22], ggpmisc [5] and doBy [11]. Graphs were produced using the packages gpplot2 [21] and gridExtra [6].

## Results

### Uptake

**Fig. 1:**
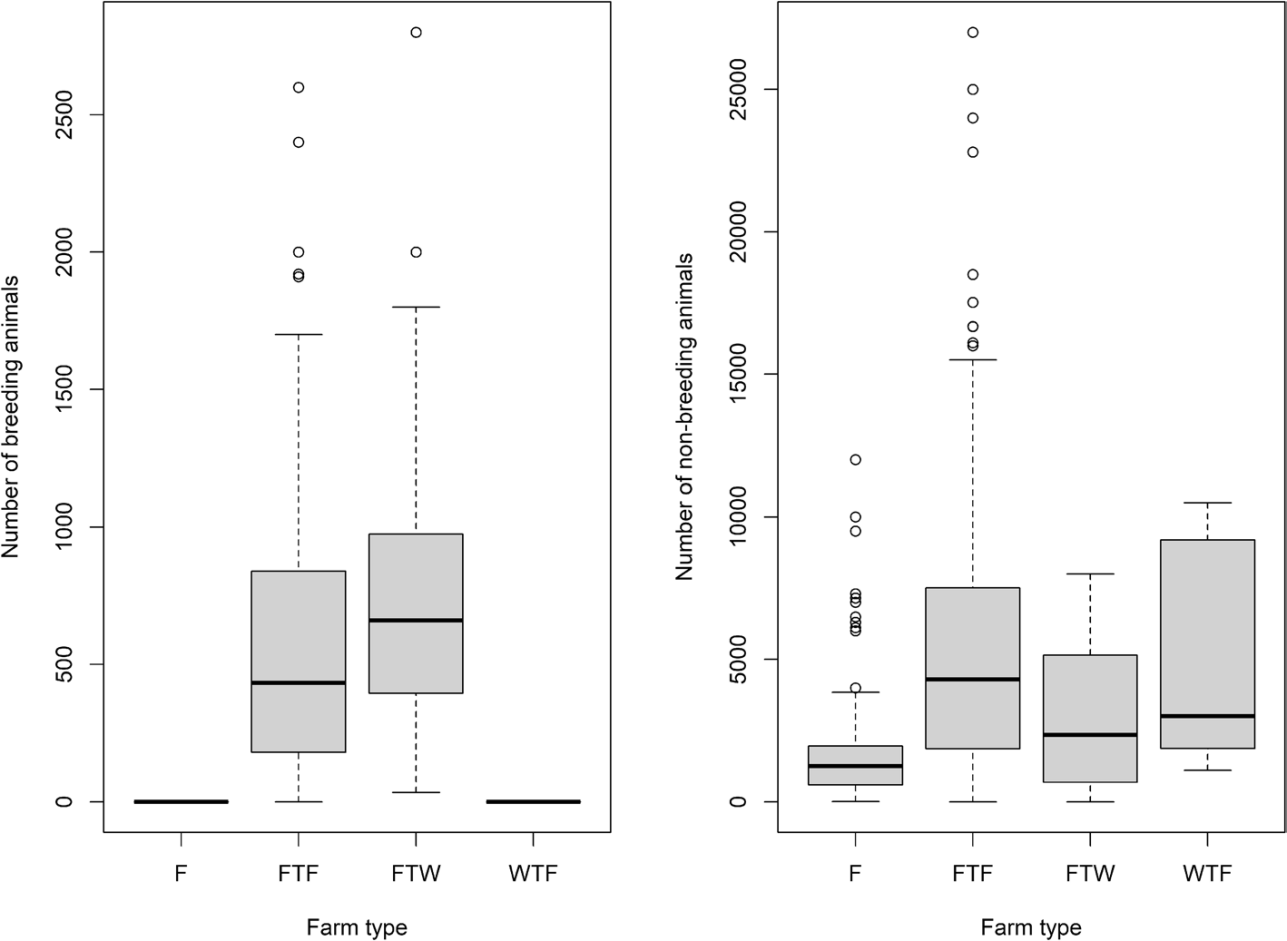
Farm size (based on the most recent assessment for each farm) for breeding and non-breeding animals per farm type. Legend: F: finisher units, FTF: farrow to finisher units, FTW: farrow to weaners units, WTF: weaner to finisher units

While a total of 16 PVPs have participated in the programme, most of the assessments have been done by only 10 PVPs. From the start of the assessments (January 2018) until December 2023, 1,014 surveys were completed on 393 pig units. The farms assessed in 2022, accounted for 92.3% and 93.8% of the breeding and non-breeding animals in Ireland, respectively, based on the Pig Census [3]. The number of assessments carried out increased from 2018 to 2022, both in terms of each production system and overall, with a decrease in 2023 (Table 1). The two most common production systems assessed were farrow-to-finish, followed by finisher units (Table 1). Total herd sizes varied between and within production systems (Fig. 1). FTF herds were the largest breeding herds, with a median of 583 breeding animals, as also the largest non-breeding herds, with a median of 5,432 animals (weaned and fattening pigs). The majority of the farms were assessed more than once from 2018 to 2023 (Table 2), and the time interval between assessments had a median of 388 days with a minimum of 60 days and a maximum of 1599 days (Supplementary Material – Table S1).

**Table 1:** Number and percentage of assessments carried out per year by production system

Year	Production system – number (%) of surveys				Total number of assessments
	Farrow to finish	Farrow-to-weaner	Weaner to finisher	Finisher	
2018	23 (82.2)	3 (10.7)	0 (0)	2 (7.1)	28
2019	53 (56.4)	7 (7.4)	7 (7.4)	26 (27.7)	94^a^
2020	81 (54.7)	11 (7.4)	5 (3.4)	51 (34.5)	148
2021	87 (50.6)	12 (7.0)	10 (5.8)	63 (37.4)	172
2022	154 (50.7)	20 (6.6)	16 (5.2)	114 (37.4)	304
2023	128 (47.8)	21 (7.8)	12 (4.5)	107 (40.0)	268

^a^one assessment had no farm type associated with it

**Table 2:** Number (%) of the 1,014 assessments carried out per farm from 2018–2023

Number of assessments done per farm during the study period (2018–2023)	Number (%) of farms
1	89 (22.6%)
2	121 (30.8%)
3	86 (21.9%)
4	61 (15.5%)
5	35 (8.9%)
6	1 (0.3%)

### Biosecurity results over time per subcategory and farm type

#### Combined scores

**Fig. 2:**
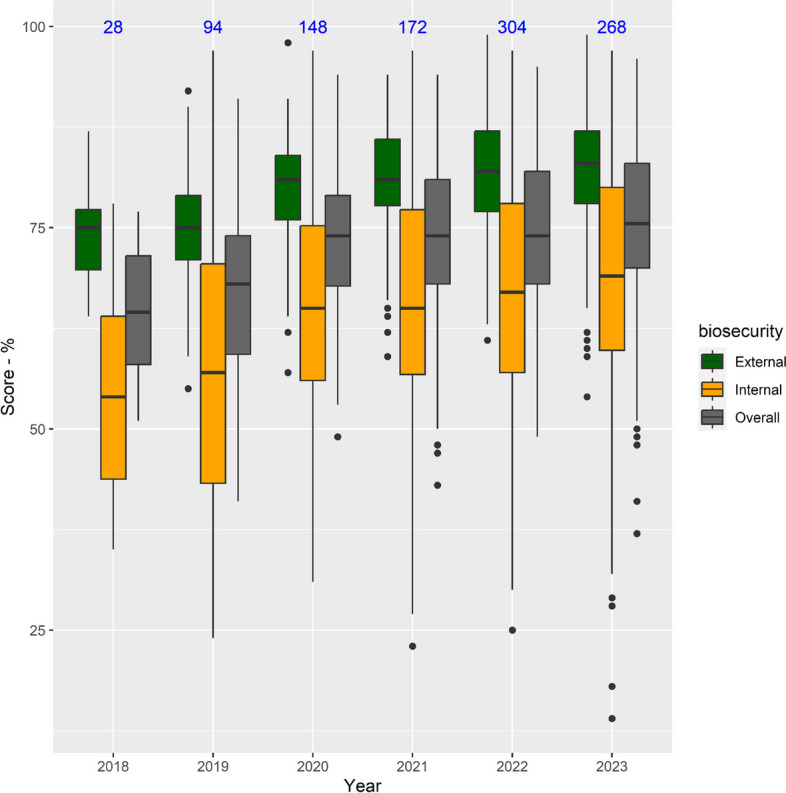
Boxplot of the distribution of the scores for external, internal and overall biosecurity from 2018 to 2023. Legend: The blue numbers are the number of farms assessed per year

External biosecurity scores were higher than internal biosecurity scores, with median scores progressively improving for both categories, and overall, between 2018–2023 (Fig. 2). However, there were wide variations in the scores (especially for internal biosecurity) between farms in each year (Fig. 2) and statistically significant differences were only observed from 2020–2023 in comparison with 2019 (Supplementary Material – Table S2). External and internal biosecurity scores per farm showed a moderate significant (*r* = 0.43, *p* < 0.001) correlation (i.e. farms with a high score for external biosecurity also tend to have a high score for internal biosecurity) (Supplementary Material – Figure S1).

#### Scores by subcategories

**Fig. 3:**
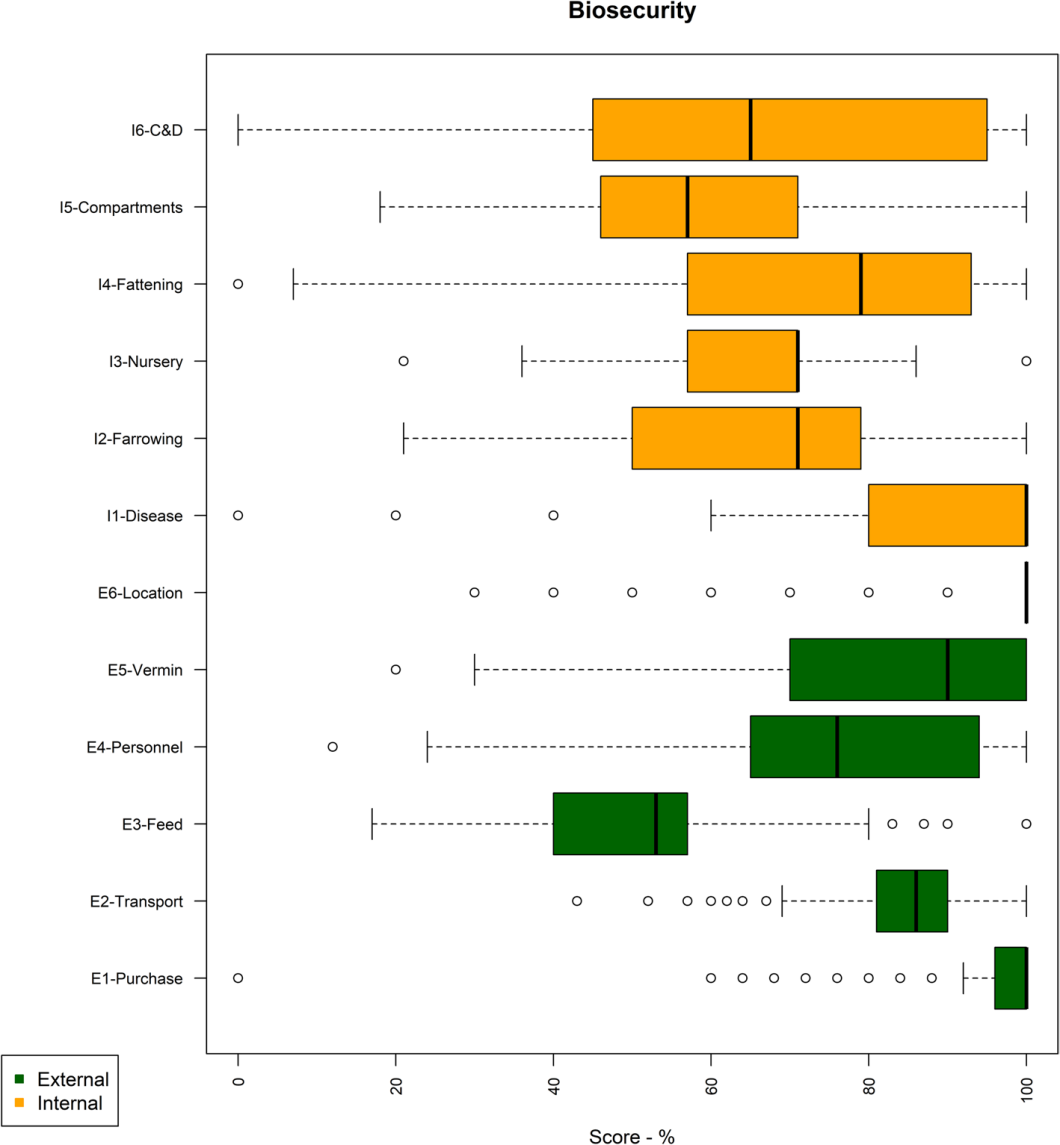
Distribution of the scores per subcategory for external (green) and internal (orange) biosecurity for the most recent results of all 393 farms that were assessed at least once

Looking in detail at the subcategories for external and internal biosecurity and considering only the most recent result per farm (Fig. 3), some of the areas with the lowest median scores are the management of *feed, water and equipment* (E3-feed) coming into the farms; the *measures implemented between compartments* (I5-Compartments) and the *farrowing unit and suckling period* (I2-Farrowing) to decrease disease transmission, and *cleaning and disinfection procedures* (I6-C&D)*,* with median scores of 53, 57, 64 and 65, respectively (Supplementary Material—Table S3). As per the combined scores, there was a wide variation of the subcategory scores between farms, especially for those related to measures implemented in the farrowing unit and sucking period and cleaning and disinfection (Fig. 3).

**Fig. 4:**
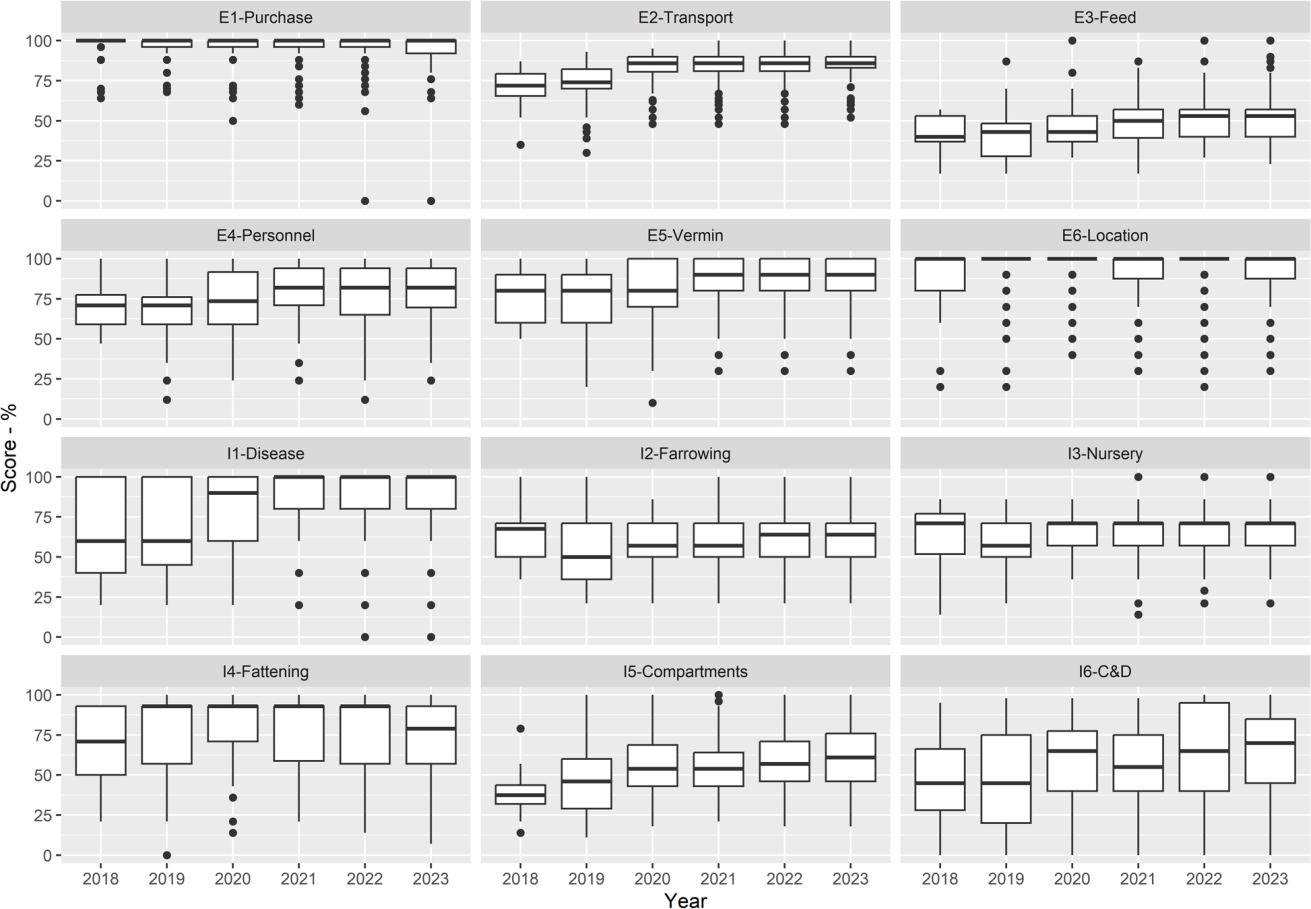
Boxplot with the distribution of the scores overtime for each subcategory of external (E) and internal (I) biosecurity. Legend: See methods section for what each subcategory means

Analysing all twelve subcategories the one that has shown the biggest improvement during the study period was *disease management* (I1-Disease; Fig. 4), followed by *cleaning and disinfection* (I6-C&D) and *measures between compartments and the use of equipment* (I5-Compartments). The other subcategories that have shown improvements over time were mainly measures related with *transport of animals*, *removal of manure and dead animals* (E2-Transport); measures associated with *personnel and visitors* (E4-Personnel); measures to control *vermin and birds* (E5-Vermin); and measures associated with *feed, water and equipment supply* (E3-Feed) (Fig. 4).

#### Scores per farm type

**Fig. 5:**
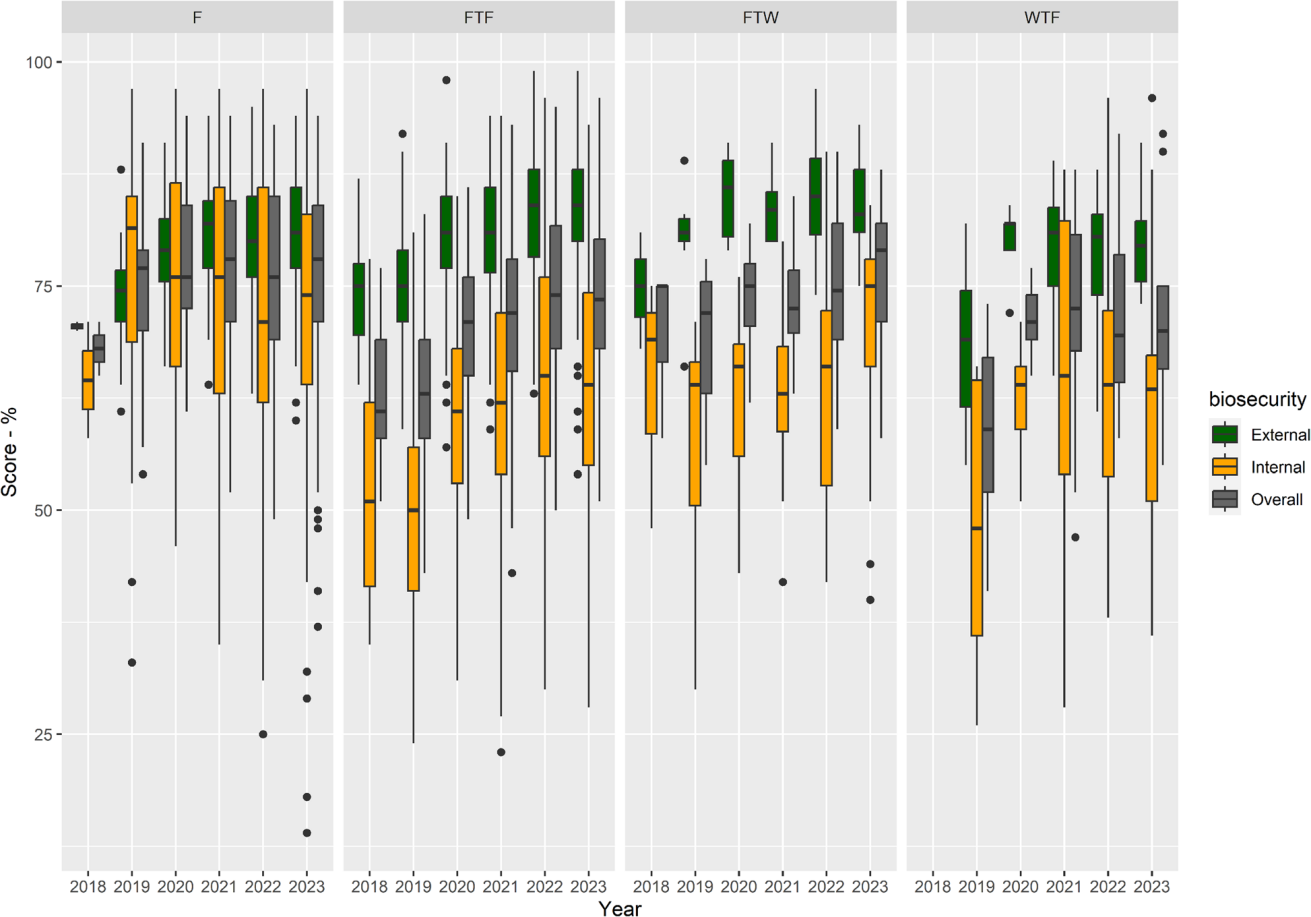
Boxplots with the distribution of scores for external, internal and overall biosecurity per production type over the years of the study. Legend: F: finisher units, FTF: farrow to finisher units, FTW: farrow to weaners units, WTF: weaner to finisher units

Weaner-to-finisher farms had lower median scores for overall and external biosecurity than the other farm types (Fig. 5, Supplementary Material—Table S4). For external biosecurity FTF and FTW farms had a higher median score than the other two farm types, while finisher farms had a higher median score for overall and internal biosecurity than the other farm types (Fig. 5). The only occasion when the median score for external biosecurity was lower than the internal score was in 2019 for the finisher-type farms. There was a statistically significant difference in the average of the internal biosecurity scores between FTF and F farms for 2019 (*p* < 0.0001), 2020 (*p* < 0.0001), 2021 (*p* < 0.0001), 2022 (*p* = 0.005) to 2023 (*p* = 0.004). Mean internal biosecurity was always better in the F farms compared to the FTF farms. Overall biosecurity scores were also statistically different between these two farm types for 2019 (p< 0.0001), 2020 (p< 0.0001) and 2021 (*p* = 0.003) years, with F farms usually getting a higher biosecurity score than FTF farms. In relation to other farm types, there were statistically significant differences between FTW and F for internal biosecurity for 2019 (*p* = 0.011) and 2020 (*p* = 0.009) – F farms with better internal biosecurity than FTW farms; WTF and F farms for internal (*p* < 0.0001) and overall (*P* = 0.0002) biosecurity scores for 2019 – F farms with better scores than WTF farms; and WTF and FTW for external biosecurity scores for 2019 (*p* = 0.017) – FTW with higher external biosecurity score than WTF.

#### Biosecurity results by farm size

The most recent results per farm were then assessed concerning the farm size. However, no apparent relationship between farm size and the biosecurity scores was observed (Supplementary Material—Figure S2).

### Results over time for a cohort of farms with at least three assessments

#### Combined and per subcategory results

**Fig. 6:**
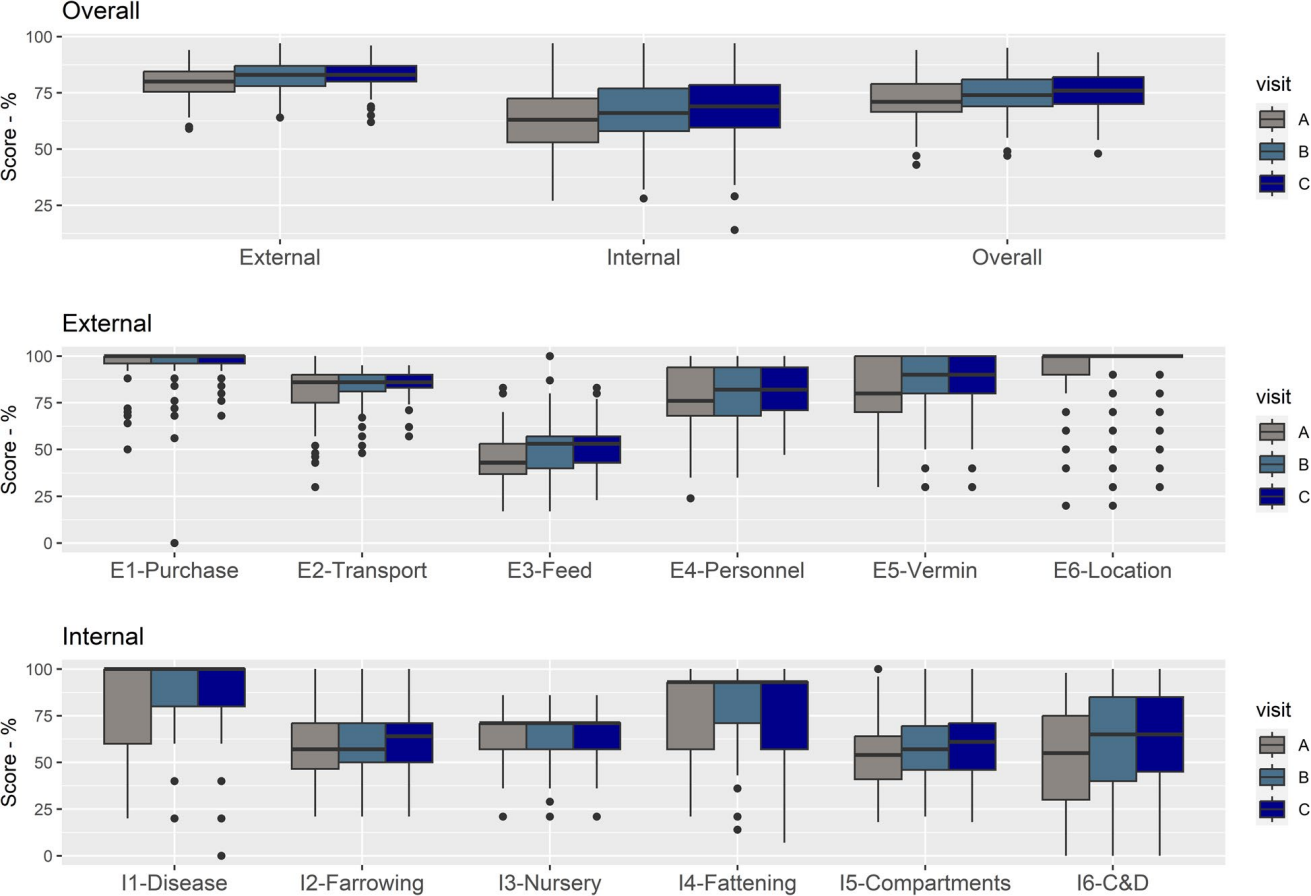
Distribution of scores for overall, external and internal biosecurity and its components for the last three most recent assessments of a cohort of 183 farms, presented in chronological order (A, B, C). Legend: See method section for what each subcategory means

Around 47% (183 – 102 farrow-to-finish farms, 14 farrow-to-weaner farms, 9 weaner-to-finisher farms and 58 finisher farms) of the farms assessed had been assessed three or more times from 2018 to 2023. Considering the most recent three assessments for this cohort of farms (and noting that these are not necessarily consecutive years), the median scores for external, internal and overall biosecurity increased from the A (the oldest) to the C (most recent) assessment (Fig. 6). For external biosecurity, this was mainly due to an increase in the scores of *measures related to feed, water and equipment supply* (E3-Feed), *personnel and visitors* (E4-Personnel), and *vermin and bird control* (E5-Vermin). For internal biosecurity, it was due to the *measures between compartments and the use of equipment* (I5-Compartments), and *cleaning and disinfection* (I6-C&D) (Fig. 6).

#### Combined scores per farm type

**Fig. 7:**
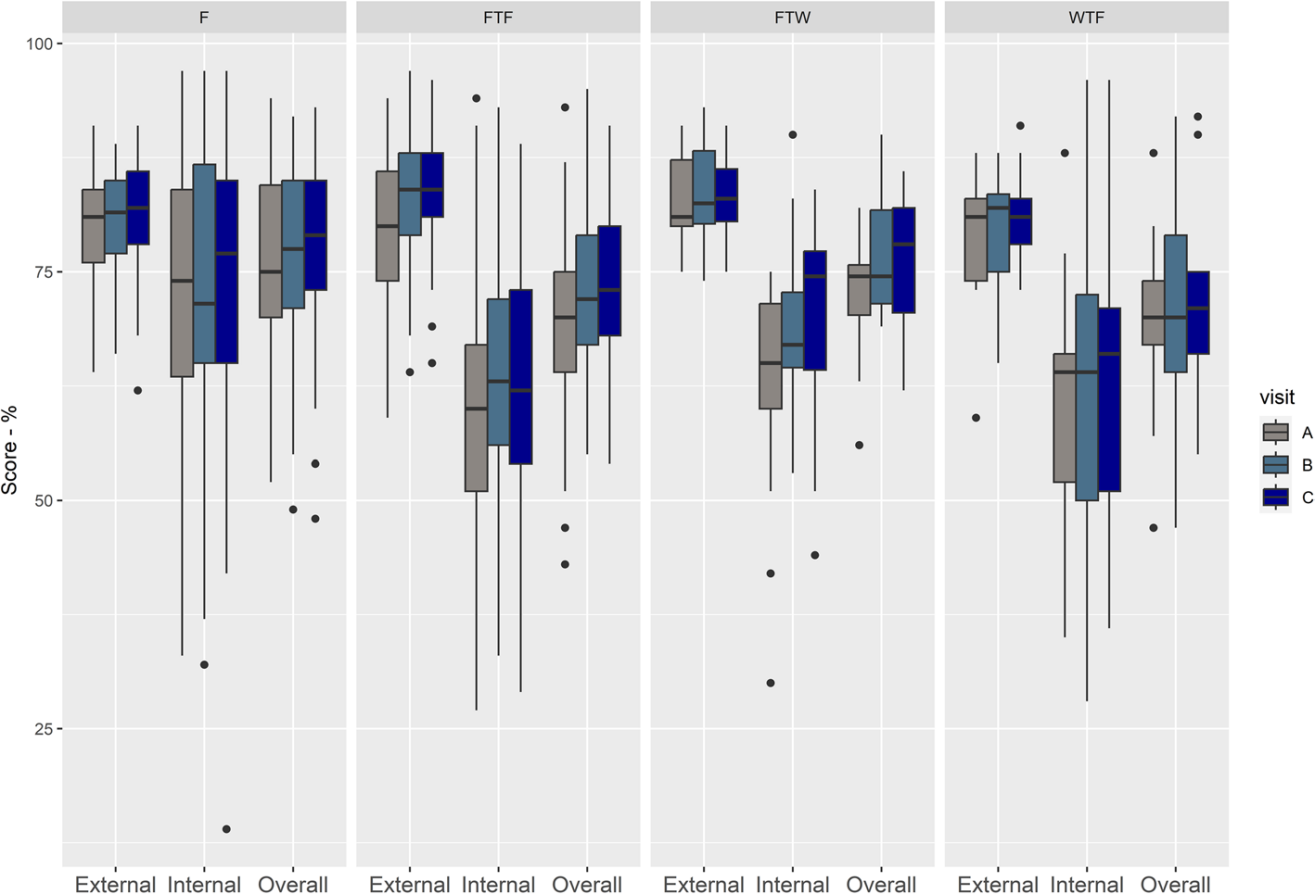
Distribution of scores for overall, external and internal biosecurity for the last three most recent assessments of a cohort of 183 farms per production type, presented in chronological order (A, B, C). Legend: F: finisher units, FTF: farrow to finisher units, FTW: farrow to weaners units, WTF: weaner to finisher units

The median overall biosecurity scores improved over time for all the farm types (Fig. 7, Supplementary Material – Table S5). Similar pattern was observed for external biosecurity, while for internal biosecurity improvements were more evident for farrow-to-weaners farms (Fig. 7).

## Discussion

In this study biosecurity scores in commercial Irish pig farms were assessed over time and by production type using a widely used biosecurity assessment system [10, 12] and areas for improvement were identified.

### Uptake

These assessments began in 2018, with farms initially participating on a voluntary basis, with the number of assessments increasing over time. In September 2021 an update to the Standard of Bord Bia (Irish Food Board – it is an Irish state agency with the aim of promoting sales of Irish food and horticulture both in Ireland and abroad) Pig Quality Assurance Scheme (PQAS) (https://www.bordbia.ie/farmers-growers/get-involved/become-quality-assured/pigmeat-quality-assurance-scheme-pqas/) included the requirement for an annual biosecurity for members. As the majority of commercial pig farms are PQAS members, that further increased engagement levels (Table 1). Furthermore, in June/July of 2022 a government-funded exceptional payment scheme for pig farmers had as one of its eligibility criteria that a Target Advisory Service for Animal Health (TASAH) Pig HealthCheck Biosecurity assessment had to have been completed between 1 January 2021 and 11 July 2022 [4]. As a result, the majority of the cohort of farms that had not previously been assessed also participated. That is why around 84% of the pig farms with more than 100 pigs (310 out of 369 farms – National Pig Census 2022 [3]) have done at least one biosecurity assessment during the study period.

### Biosecurity levels

External biosecurity scores higher than internal biosecurity mainly due to the weight that is attributed to the purchase of animals and semen. As most Irish farms follow a farrow-to-finish production type (closed cycle) and raise their own replacement stock, very few breeding animals are purchased (only for genetic improvement). Furthermore, the vast majority of the farms use semen from sources with high health status. This way, the risk of introducing new diseases through the purchase of replacement animals and semen is much reduced.

All farms had higher external biosecurity scores than internal scores, and the median scores showed improvement between the years 2018 and 2023 (Figs. 2 and 4), except for finisher farms in 2019. However, there was considerable variability in the number of farms assessed over the years, in the scores between farms (Fig. 2), within subcategories (Fig. 3), with the variability in scores for the management of *cleaning and disinfection* being greatest. This probably reflects differences between farms in terms of infrastructure and/or management (including farmer and staff attitudes in relation to biosecurity). Further research would be needed to explore these differences between farms. C*leaning and disinfection* and *measures between compartments and the use of equipment* are the subcategories that contribute more to the internal biosecurity score (https://biocheckgent.com/en/weight-factors-pig), reflecting the importance of the measures contained within these subcategories to decrease the infection cycle within a farm [10]. When looking at the results over time (Figs. 4 and 6) these two subcategories are improving, indicating that the current system is working by flagging these as issues to improve and that efforts have been made by farmers to address them. Practices showing improving scores over time in these subcategories are the cleaning and disinfection of the rooms after each production cycle, washing hands between different compartments, and using designated equipment for each room (data not shown).

While measures related to the *fattening unit* showed improvement over the years*,* the 2023 result was a step back from previous years. This is considered to reflect the increase in overstocking at the fattening stage in 2023 compared to 2022 (data not shown).

Overall, the areas identified for improvement for internal biosecurity were *nursery unit*, *farrowing unit and suckling period* and *measures between compartments and the use of equipment*. These are the subcategories with the lowest scores, mainly because of cross-fostering practices, lack of strict all-in-all-out management, high stocking densities, lack of footbath/booth washers between the different compartments (data not shown). Internal biosecurity is especially important to break the cycle of disease transmission within a farm and to control endemic diseases (such as *E. coli*, *Salmonella spp*., etc.) [10]. Therefore, more attention is required from farmers in these areas, albeit some of these areas required more investment such as new accommodation to reduce stocking densities or a reduction in the overall number of pigs on the farm.

The *feed, water and equipment supply* subcategory corresponds to the only external subcategory with a low score. This represents a way for the introduction of diseases through contaminated feed and water, infected material, and contaminated feed lorries. The lower scores on this subcategory are mainly due to poor management of water quality and location of feed silos (which means that feed lorries have access to the areas of the production site which should be restricted) (data not shown).

The biosecurity scores also varied per farm type (Figs. 5 and 7). Farrow-to-finisher and farrow-to-weaner farms showed better external biosecurity scores than the other farm types. However, their internal biosecurity scores were poorer compared to finisher farms. This probably reflects the complexity of managing all or almost all stages of pig production on the same farm, especially when managing the internal biosecurity, i.e., the measures within the farm. Finisher farms were the only type of farm where there was the least difference between external and internal biosecurity scores. This is probably due to this type of farm being easier to manage (i.e. only one stage/compartment of the pig production, and lower number of animals per farm).

In 2022 the Irish pig industry experienced its lowest profitability in 40 years as the invasion of Ukraine led to escalating feed ingredient and energy costs [7]. The situation in 2023 improved but the accumulated cashflow losses from that period (end of 2021 to the beginning of 2023) had not yet been recovered [7]. Even so, the biosecurity scores for 2022 and 2023 have overall been maintained or increased, showing that there were still “low-hanging fruit” biosecurity practices that could be implemented. However, in the future, further gains in biosecurity may require capital investments (e.g. new buildings with better layout to separate high-risk procedures from low-risk procedures or easier to clean and disinfect) or extra labour, which the sector might not be able to afford (due to lack of financial resources or difficult in sourcing staff) in the short term. Therefore, it is essential to demonstrate to farmers the cost–benefit of implementing different biosecurity measures so that information can help their decision-making process for better returns on their investments. The data obtained through this study will be used for those cost–benefit estimations.

### Strengths and weaknesses of the biosecurity assessments

The current system to assess biosecurity in pig farms has some limitations as it uses different assessors and, albeit they have been trained, that introduces an element of variability as different assessors do bring their own subjectivity when interpreting the questions within the assessment. Furthermore, the assessors are the farms’ veterinarians and therefore have some degree of connection with the farmers that might impair them from an independent assessment of the farms. The median time interval between consecutive assessments at farm level was around one year, however with a wide variation, and for some farms it went to the extreme of 4 years between assessments. This is not conducive to supporting the farmer when they start implementing recommended practices, as most of the time some adjustments are necessary to ensure an efficient implementation and the different time interval between assessments might have affected the farm score. However, using the farm’s veterinarian as the biosecurity assessor also brings some advantages, as it overcomes the limitation of the frequency of assessments as the farm’s veterinarian will be visiting the farm frequently for other reasons (including work in other areas of the PHC programme) and can then support the farmer on the implementation of biosecurity measures [9, 13]. Moreover, the farm’s veterinarian is familiar with the farm (including health status), the farmer and workers and therefore can co-design with the farmer the most appropriate biosecurity practices to implement on the farm and this way ensure their effective implementation. Uptake and effective implementation of the biosecurity recommendations will improve biosecurity in the first instance at farm level and then at national level. A good working relationship between the farm’s veterinarian and the farmer will enable this.

## Conclusions

Through the continued efforts of the Irish pig industry and their nominated veterinary practitioners, with government support, the last few years have registered improvements in the biosecurity scores of the farms. Although external biosecurity is considered high, internal biosecurity has room for improvement and with this will prevent disease spread within the units and consequently reduce disease prevalence for endemic diseases such as salmonellosis, PRRS, colibacillosis and other common pathogens bringing additional economic benefits and improving the overall scores and performances of the farms [19].

## Supplementary Information

Supplementary Material 1.

## Data Availability

Aggregated data is available from first author at a reasonable request.

## Abbreviations

FTF: Farrow-to-weaner
WTF: Weaner-to-finisher
FTW: Farrow-to-weaner
F: Finisher
PRRS: Porcine reproductive and respiratory syndrome
TASAH: Targeted Advisory Service on Animal Health
PVPs: Private veterinary practitioners
AHI: Animal Health Ireland
API: Application programming interface
