# Supplementary material for: Temporal trends in biosecurity in Irish pig herds using a standardized scoring system
Source: Ir Vet J. 2025 Jan 30;78:4. doi: 10.1186/s13620-025-00289-0 (PMC11780990; doi:10.1186/s13620-025-00289-0)
Supplement: Supplementary file 1 — Supplementary Material 1. [file 13620_2025_289_MOESM1_ESM.docx]

**Supplementary material**

Table S1 – Summary statistics of the time interval (in days) between assessments in the same farms

| Time interval between assessments | Minimum | Median | Mean | Maximum |
| --- | --- | --- | --- | --- |
| First to second | 60 | 444 | 550.2 | 1599 |
| Second to third | 122 | 381 | 426.4 | 937 |
| Third to fourth | 134 | 357 | 373.6 | 827 |
| Fourth to fifth | 184 | 328 | 346.5 | 607 |
| Fifth to sixth | 184 | 184 | 184 | 184 |
| All time intervals – all farms | 60 | 388 | 473.2 | 1599 |
| All time intervals – cohort of 183 farms | 98 | 384 | 473.5 | 1485 |

| Table S2 – Summary of the mean differences and Turkey post hoc p value (p) of the combined scores over the years Year comparison | External | | Internal | | Overall | |
| --- | --- | --- | --- | --- | --- | --- |
|  | Mean difference | p | Mean difference | p | Mean difference | p |
| 2020-2019 | 5.15 | <0.0001 | 8.00 | 0.001 | 6.60 | <0.0001 |
| 2021-2019 | 5.98 | <0.0001 | 8.22 | 0.0004 | 7.14 | <0.0001 |
| 2022-2019 | 6.97 | <0.0001 | 9.51 | <0.0001 | 8.24 | <0.0001 |
| 2023-2019 | 7.30 | <0.0001 | 10.28 | <0.0001 | 8.82 | <0.0001 |
| 2021-2020 | 0.83 | 0.825 | 0.22 | 0.999 | 0.55 | 0.987 |
| 2022-2020 | 1.82 | 0.073 | 1.51 | 0.869 | 1.64 | 0.444 |
| 2023-2020 | 2.15 | 0.022 | 2.28 | 0.607 | 2.22 | 0.164 |
| 2022-2021 | 0.99 | 0.580 | 1.30 | 0.908 | 1.09 | 0.765 |
| 2023-2021 | 1.32 | 0.300 | 2.06 | 0.655 | 1.67 | 0.391 |
| 2023-2022 | 0.33 | 0.979 | 0.76 | 0.977 | 0.58 | 0.952 |

Table S3 – Summary statistics of the biosecurity scores for all farms assessed in 2023 (268 farms)

| Biosecurity subcategory | Minimum | Q1 | Median score | Mean | Q3 | Maximum |
| --- | --- | --- | --- | --- | --- | --- |
| Internal |  |  |  |  |  |  |
| A. Disease management | 0 | 80 | 100 | 86 | 100 | 100 |
| B. Farrowing unit and suckling period | 21 | 50 | 64 | 63 | 79 | 100 |
| C. Nursery unit | 21 | 57 | 71 | 66 | 71 | 100 |
| D. Fattening unit | 0 | 57 | 79 | 75 | 93 | 100 |
| E. Measures between compartments and the use of equipment | 18 | 46 | 57 | 62 | 71 | 100 |
| F. Cleaning and disinfection | 0 | 45 | 65 | 63 | 95 | 100 |
| External |  |  |  |  |  |  |
| A. Purchase of animals and semen | 0 | 92 | 100 | 94 | 100 | 100 |
| B. Transport of animals, removal of manure/dead animals | 43 | 81 | 86 | 86 | 90 | 100 |
| C. Feed, water and equipment supply | 17 | 40 | 53 | 53 | 57 | 100 |
| D. Personnel and visitors | 12 | 65 | 76 | 78 | 94 | 100 |
| E. Vermin and bird control | 20 | 70 | 90 | 84 | 100 | 100 |
| F. Environment and region | 30 | 100 | 100 | 93 | 100 | 100 |

Table S4 – Median of the biosecurity scores for all farms assessed during the study period per farm type

| Year of assessment | Biosecurity | Finisher farms | Farrow-to-finisher farms | Farrow-to-weaner farms | Weaner-to-finisher farms |
| --- | --- | --- | --- | --- | --- |
| 2018 | Overall | 68 | 62.5 | 75 | - |
|  | External | 70.5 | 75 | 75 | - |
|  | Internal | 64.5 | 52 | 69 | - |
| 2019 | Overall | 77 | 62.5 | 72 | 59 |
|  | External | 74.5 | 75 | 81 | 69 |
|  | Internal | 81.5 | 50 | 64 | 48 |
| 2020 | Overall | 76 | 71 | 75 | 71 |
|  | External | 79 | 81 | 86 | 82 |
|  | Internal | 76 | 61 | 66 | 64 |
| 2021 | Overall | 78 | 72 | 72.5 | 71 |
|  | External | 82 | 81 | 83.5 | 81 |
|  | Internal | 76 | 62 | 63 | 64 |
| 2022 | Overall | 76 | 74 | 74.5 | 70 |
|  | External | 80 | 84 | 85 | 81 |
|  | Internal | 71 | 65 | 66 | 64 |
| 2023 | Overall | 78 | 73.5 | 79 | 70 |
|  | External | 81 | 84 | 83 | 79.5 |
|  | Internal | 74 | 64 | 75 | 63.5 |

Table S5 – Median of the biosecurity scores for last three most assessments (presented in chronological order (A, B, C)) for the cohort of farms with at least three assessments during the study period per farm type

| Assessment | Biosecurity | Finisher farms | Farrow-to-finisher farms | Farrow-to-weaner farms | Weaner-to-finisher farms |
| --- | --- | --- | --- | --- | --- |
| A | Overall | 75 | 70 | 74.5 | 70 |
|  | External | 81 | 80 | 81 | 81 |
|  | Internal | 74 | 60 | 65 | 64 |
| B | Overall | 77.5 | 72 | 74.5 | 70 |
|  | External | 81.5 | 84 | 82.5 | 82 |
|  | Internal | 71.5 | 63 | 67 | 64 |
| C | Overall | 79 | 73 | 78 | 71 |
|  | External | 82 | 84 | 83 | 81 |
|  | Internal | 77 | 62 | 74.5 | 66 |


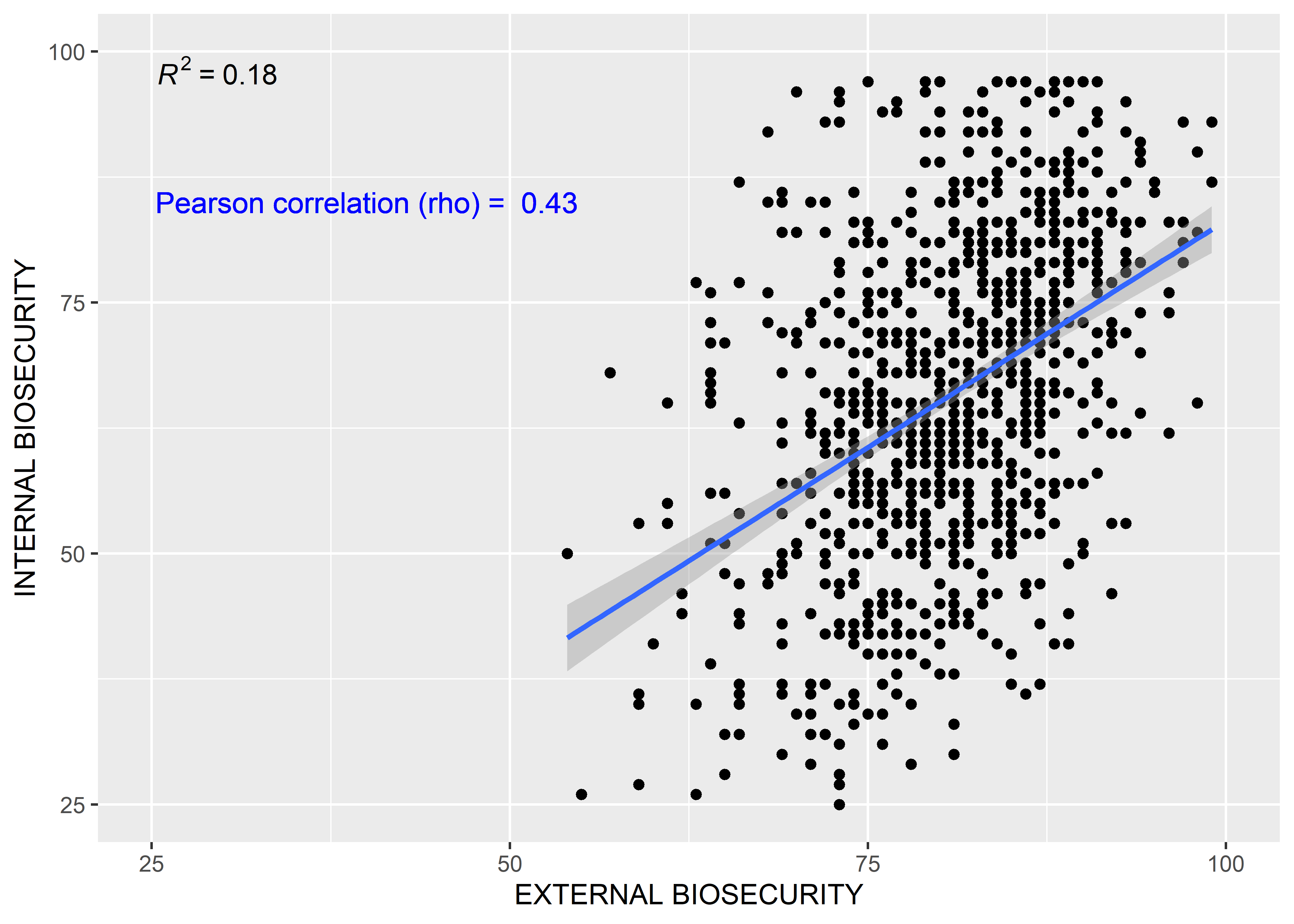


Figure S1: Distribution of the scores per farm for external and internal biosecurity for all biosecurity surveys for all 393 farms that were assessed at least once, including fitted line, coefficient of determination (R^2^) and correlation coefficient.


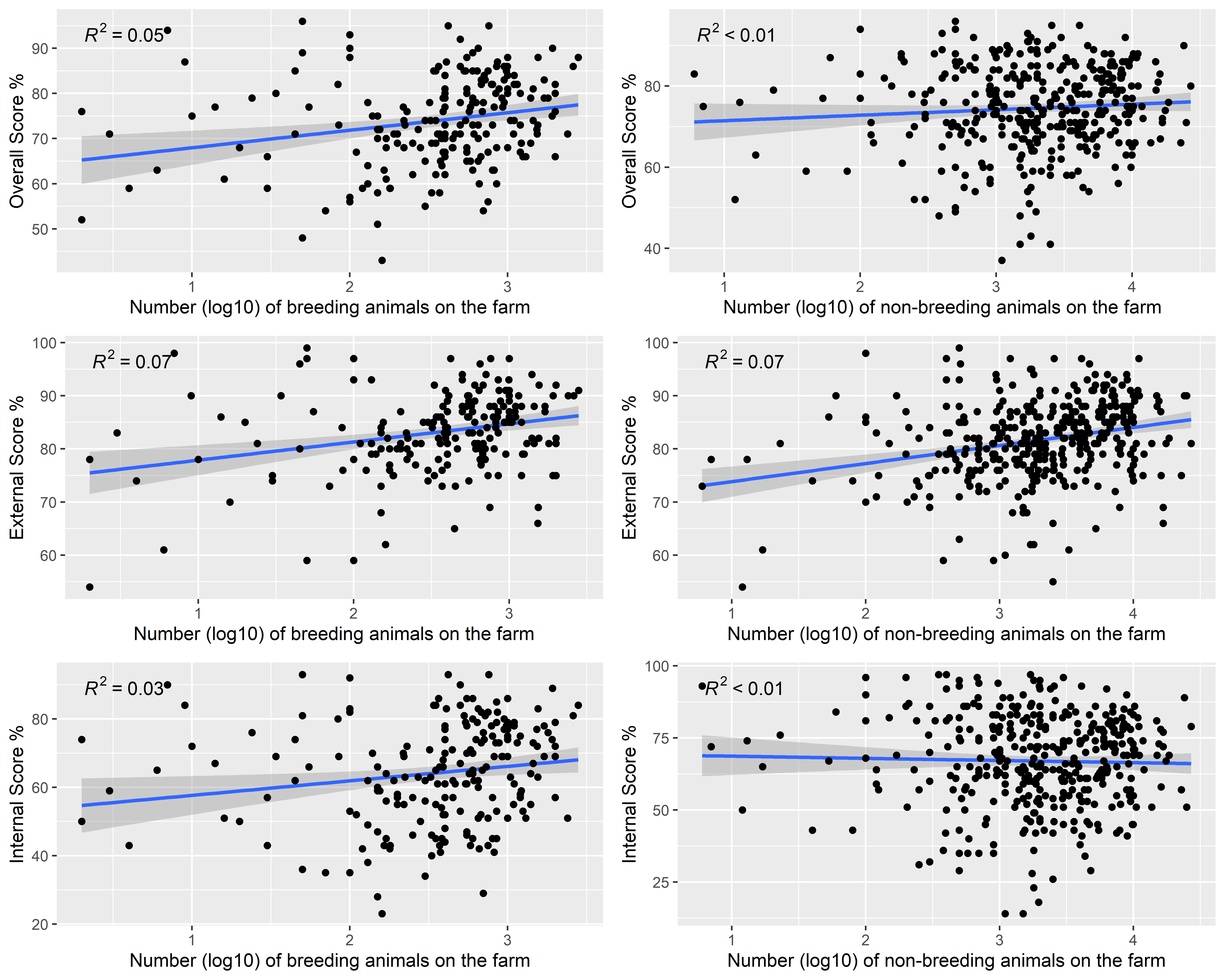


Figure S2: Biosecurity scores (overall (first row), external (second row) and internal (third row)) by size of the farm in log10 scale (breeding animals left panel, non-breeding animals right panel).
